# Spectroscopic Probing of Solute–Solvent Interactions in Aqueous Methylsulphonylmethane (MSM) Solutions: An Integrated ATR-FTIR, Chemometric, and DFT Study

**DOI:** 10.3390/ijms262210953

**Published:** 2025-11-12

**Authors:** Aneta Panuszko, Przemysław Pastwa, Paulina Giemza, Piotr Bruździak

**Affiliations:** Department of Physical Chemistry, Gdańsk University of Technology, Narutowicza 11/12, 80-233 Gdańsk, Poland; aneta.panuszko@pg.edu.pl (A.P.);

**Keywords:** methylsulphonylmethane (MSM), ATR-FTIR spectroscopy, optical artefacts, chemometrics, DFT calculations

## Abstract

The widespread use of methylsulphonylmethane (MSM) as a dietary supplement highlights the need to understand its fundamental behaviour in aqueous solutions. In this paper, we investigate changes in the MSM band shape as a function of its concentration using Attenuated Total Reflection FTIR (ATR-FTIR) spectroscopy. ATR spectra may be complicated by significant optical artefacts arising from refractive index changes. These can be misinterpreted as genuine vibrational shifts, leading to erroneous conclusions. Here, we systematically investigate aqueous MSM solutions using three different internal reflection elements. Applying a rigorous ATR correction procedure, validated by transmission measurements and PARAFAC (Parallel Factor Analysis) analysis, decouples physical phenomena from optical distortions. The corrected spectra reveal a crucial finding: the primary effect of MSM is not a shift in the sulphone band position, but a distinct change in its shape. This result, supported by DFT (Density Functional Theory) calculations, indicates increased heterogeneity of local hydration environments and demonstrates the criticality of proper ATR correction.

## 1. Introduction

Methylsulphonylmethane (MSM), the oxidised form of dimethyl sulphoxide (DMSO) and also known as dimethyl sulphone (DMSO_2_), is a well-known organosulphur compound. It has gained widespread popularity as a dietary supplement [1] and plays an important role in the treatment of conditions related to bones and joints [2,3,4]. It is believed to act as a sulphur donor for the synthesis of crucial biomolecules like the amino acids methionine and cysteine, thus supporting the integrity of connective tissues such as collagen and keratin [5]. Moreover, MSM has been linked to antioxidant properties through the modulation of oxidative stress pathways [1], and recent studies have highlighted its potential in sensitising cancer cells to chemotherapeutic agents [6]. These emerging biological roles further underscore the importance of understanding MSM’s molecular interactions in a physiologically relevant aqueous environment.

The chemical and physical properties of methylsulphonylmethane (MSM) have been extensively characterised, primarily in the solid state and gas phase, using techniques such as X-ray diffraction [5], infrared and Raman spectroscopy [7], and NMR spectroscopy [8]. Computational studies have often focused on the properties of the isolated molecule or its small clusters in non-bulk environments [9,10]. While some research has begun to probe its behaviour in bulk solution using methods like dielectric relaxation spectroscopy [11] and to explore its hydration shell using a combination of infrared spectroscopy and ab initio molecular dynamics (AIMD) simulations [12], a comprehensive, molecular-level picture of its aqueous interactions remains surprisingly limited. This knowledge gap is particularly significant because water is the fundamental medium for all biological activity. Consequently, this limits a deeper understanding of MSM’s mechanisms of action and hinders the development of robust and sensitive methods for its quantification in complex biological or pharmaceutical systems.

Among various analytical techniques, Attenuated Total Reflection Fourier-Transform Infrared (ATR-FTIR) spectroscopy stands out as a particularly promising tool for the label-free analysis of solutes in aqueous solutions. It allows for the direct analysis of liquid samples with minimal preparation and provides rich information about molecular structure and interactions [13,14,15]. However, to leverage ATR-FTIR for quantitative analysis, it is imperative to first address and eliminate potential optical artefacts that can distort spectral features. The ATR measurement principle inherently introduces a wavelength-dependent depth of penetration of the evanescent wave, and changes in the sample’s refractive index can cause shifts and intensity variations in absorbance bands. These artefacts can be easily misinterpreted as genuine chemical phenomena, such as changes in hydrogen bonding or conformation, leading to incorrect conclusions about the system under study. This risk is particularly acute in concentration-dependent studies, as it is often assumed that for aqueous solutions with only minor variations in refractive index, the need for a robust ATR correction is negligible [14]. This work directly challenges that assumption and demonstrates that for such studies, applying a validated ATR correction is not optional but mandatory to distinguish true molecular phenomena from measurement artefacts [15]. Therefore, a central focus of this study is to underscore the necessity of a validated correction procedure to prevent the erroneous reporting of optical effects as genuine molecular interactions, a topic of ongoing methodological advancement [15].

The primary objective of this study is, therefore, to establish a validated spectroscopic framework for analysing MSM in aqueous solutions that can distinguish true solvation effects from measurement artefacts. We systematically investigate the FTIR spectra of MSM over a wide concentration range using three different ATR internal reflection elements (IREs): Germanium (Ge), Zinc Selenide (ZnSe), and diamond. By comparing uncorrected and corrected spectra with “gold standard” transmission measurements and analysing the data with PARAFAC chemometrics, we identify the sources of spectral variation. Our findings are further supported by Density Functional Theory (DFT) calculations, which provide a theoretical model of MSM’s behaviour in solution. This work lays the essential groundwork for the future development of reliable ATR-FTIR-based analytical methods for MSM, ensuring that future methods based on this technology measure true biochemical phenomena rather than deceptive optical artefacts. In addition, this approach may serve as a blueprint for studying other small, water-soluble bioactive compounds with subtle spectroscopic signatures.

## 2. Results and Discussion

### 2.1. ATR-FTIR Spectra of MSM: Artefacts Versus Reality

The fingerprint region of the MSM FTIR spectrum contains three strong bands, identified by DFT calculations as asymmetric SO_2_ stretching (∼1285 cm^−1^), symmetric SO_2_ stretching (∼1138 cm^−1^), and coupled CH_2_ and SO_2_ wagging (∼950 cm^−1^).

**Figure 1:**
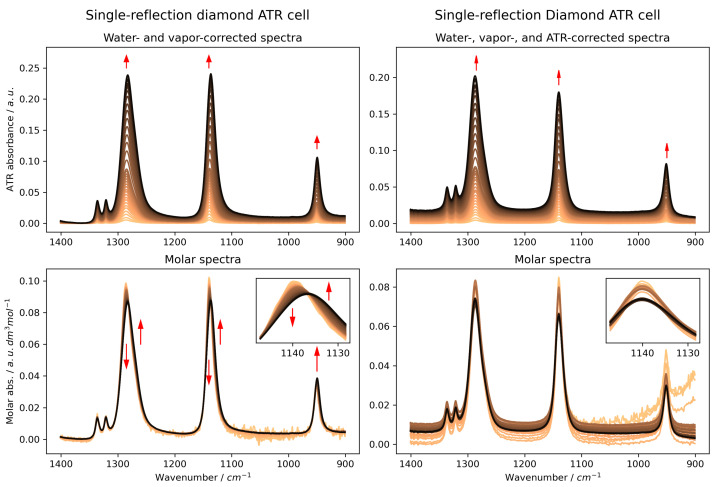
Uncorrected (**Left panels**) and ATR-corrected (**right panels**) ATR-FTIR spectra of MSM in aqueous solutions (0.0–3.0 mol·dm^−3^, no ATR correction), acquired using a single-reflection diamond ATR cell. **Upper panels**: spectra of MSM, devoid of water and water vapour contributions, in the range of SO_2_ stretching bands. **Lower panels**: concentration-corrected molar spectra of MSM. **Insets**: an enlarged fragment of the SO_2_ band at 1140 cm^−1^. Please refer to Supplementary Materials for figures corresponding to Ge and ZnSe IREs, i.e., Figures S1–S4, respectively. Arrows indicate the main changes in band shapes.

**Figure 2:**
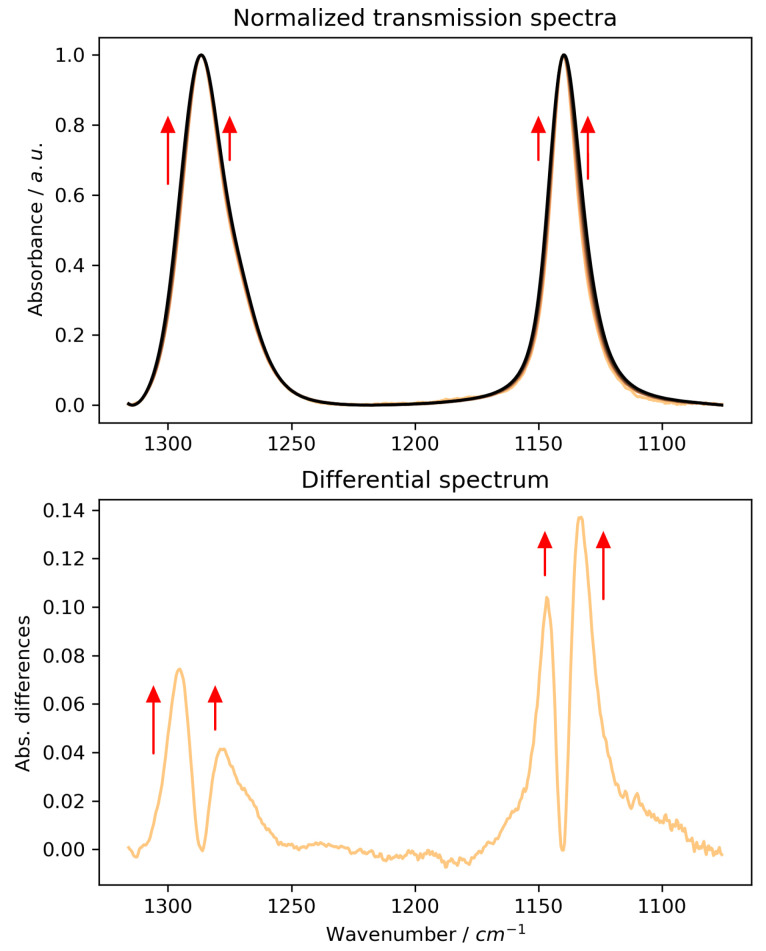
**Upper panel:** Transmission spectra of MSM solutions in the SO_2_ vibration range (0.0–3.0 mol·dm^−3^). The common minimum and maximum are used to normalise the spectrum. Red arrows are only for comparison; they show changes in normalised spectra. Using a spacer-less cuvette prevents information about sample thickness from being obtained, which makes it impossible to pinpoint the precise information on exact changes. **Lower panel:** The difference between the last and first spectra in the series. The visible pattern of difference bands, marked with arrows (their lengths correspond to changes in intensity), shows changes in the width and potential increase in the high- and low-energy sides of the symmetric and asymmetric bands, respectively.

When analysing the concentration-dependent series of uncorrected ATR spectra (Figure 1, left panels, and Figures S1 and S2 in Supplementary Materials for Ge and ZnSe, respectively), a consistent red-shift in all major bands is observed, regardless of the IRE used. This apparent shift, being more pronounced for ZnSe and diamond, could be mistakenly interpreted as a change in the hydrogen bonding environment of the sulphone group with increasing MSM concentration. To discern whether these spectral changes reflect true molecular interactions or are merely measurement artefacts, we compared them to transmission spectra (Figure 2). A more detailed discussion of the initial spectral observations and the role of the materials’ refractive indices is provided in the Supplementary Materials (see Section S2.2). The transmission data, while not quantitative due to unknown pathlength, do not show a similar systematic band shift. Instead, they clearly suggest a broadening of the SO_2_ bands with increasing MSM concentration, as seen by the growth of the high- and low-energy sides of the bands in the differential spectrum (Figure 2, lower panel).

This discrepancy strongly suggests that the red-shifts observed in uncorrected ATR spectra are primarily optical artefacts arising from the changing refractive index of the solutions. After applying the necessary ATR correction (Figure 1, right panels, and Figures S3 and S4 in Supplementary Materials), a more accurate picture of MSM’s behaviour emerges. The artificial red-shift disappears, particularly for diamond and Ge IREs, and the dominant effect of increasing MSM concentration is a broadening of the bands, not a shift in their position. This observation is now consistent with the transmission spectra and suggests that the interactions in solution lead to a greater variety of vibrational states rather than a systematic weakening or strengthening of a specific bond. This increased variety of states is the source of the observed band broadening.

### 2.2. Chemometric and Theoretical Insights into MSM Solvation

**Figure 3:**
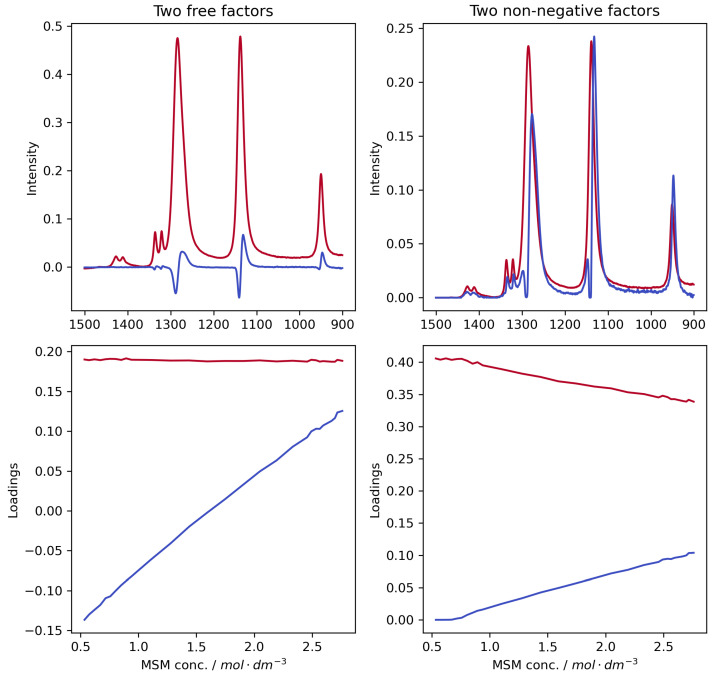
Results of PARAFAC decomposition of ATR-FTIR spectra acquired with diamond IRE. We obtained two factors (red and blue lines) and their loadings using the free, or unconstrained (**left** panels), and non-negative, or constrained, variants of the decomposition algorithm (**right** panels). Similar decomposition results for Ge and ZnSe IREs can be found in Supplementary Materials (Figures S5 and S6, respectively).

To quantitatively extract the underlying spectral components, we employed PARAFAC analysis. A detailed description of the chemometric strategy and the full analysis of both uncorrected and corrected spectra can be found in the Supplementary Materials (see Section S2). The decomposition of uncorrected spectra (Figure 3, and Figures S5 and S6 in Supplementary Materials for Ge and ZnSe) yields a derivative-like factor that perfectly describes the artefactual band shift, confirming that the observed changes are dominated by optical effects.

A closer inspection of the results for different IREs, detailed in the Supplementary Materials (Figures S1–S8), reveals the crucial role of the crystal material. The correction algorithm performs most effectively for diamond and Ge IREs, where the artefactual red-shift is almost entirely eliminated. In contrast, for the ZnSe IRE, the correction proves to be insufficient; the corrected spectra still retain some features of the uncorrected data, suggesting that ZnSe is a less suitable choice for such concentration-dependent studies. This observation underscores that the success of ATR correction is not universal and depends heavily on the specific optical properties of both the sample and the IRE.

**Figure 4:**
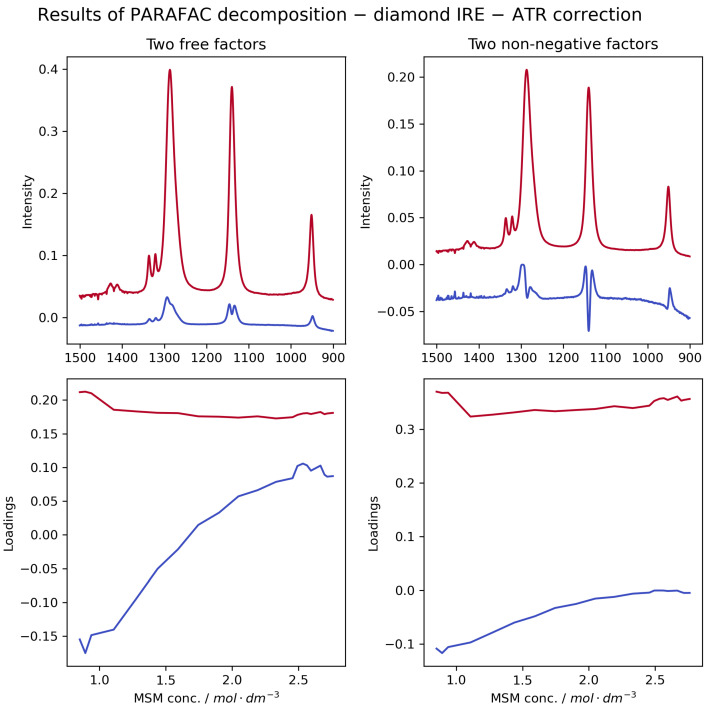
Results of PARAFAC decomposition of ATR-FTIR spectra from Figure 3 corrected with an advanced ATR correction algorithm. As shown in Figure 3, two factors (red and blue lines) and their loadings were found using both free (**left** panels) and non-negative (**right** panels) versions of the decomposition algorithm. In the right panels, we have reversed the signs of the second factor and its loading (blue) to better compare with the results of free decomposition. Such a change has only a visual meaning and gives no net change in the mathematical meaning of the blue factor. Similar decompositions for Ge and ZnSe IREs can be found in Supplementary Materials (Figures S7 and S8, respectively).

In stark contrast, analysis of the ATR-corrected spectra (Figure 4, and Figures S7 and S8 in Supplementary Materials for Ge and ZnSe) reveals the true nature of the changes, as detailed in Section S2.3 of the Supplementary Materials. Specifically, the second factor derived from chemometric analysis now reflects a change in the width of the SO_2_ bands. This indicates that as the concentration of MSM increases, the local environment around the solute molecules becomes more disordered and heterogeneous. Our previous study [12] revealed the complex structure of MSM’s hydration shell. We demonstrated that it comprises at least two distinct populations of water molecules: a larger fraction weakly hydrogen-bonded to the sulphonyl group, and a smaller, more structured fraction organised around the hydrophobic methyl groups. The symmetric broadening of the SO_2_ vibrational bands observed in the present study provides a direct spectroscopic manifestation of this heterogeneity. As the concentration of MSM increases, the local water structure is perturbed, and the environment around each sulphonyl group becomes increasingly diverse. This environment encompasses not only interactions with water molecules in various states of order but also growing interactions with neighboring MSM molecules. This variety of local environments leads to a distribution of vibrational frequencies, which manifests in the spectrum as band broadening, rather than a unidirectional shift.

Thus, the present experimental results, obtainable only through a combination of a suitable IRE and a validated correction algorithm, not only eliminate measurement artefacts but also provide strong evidence for the previously proposed complex model of MSM hydration at the molecular level.

**Figure 5:**
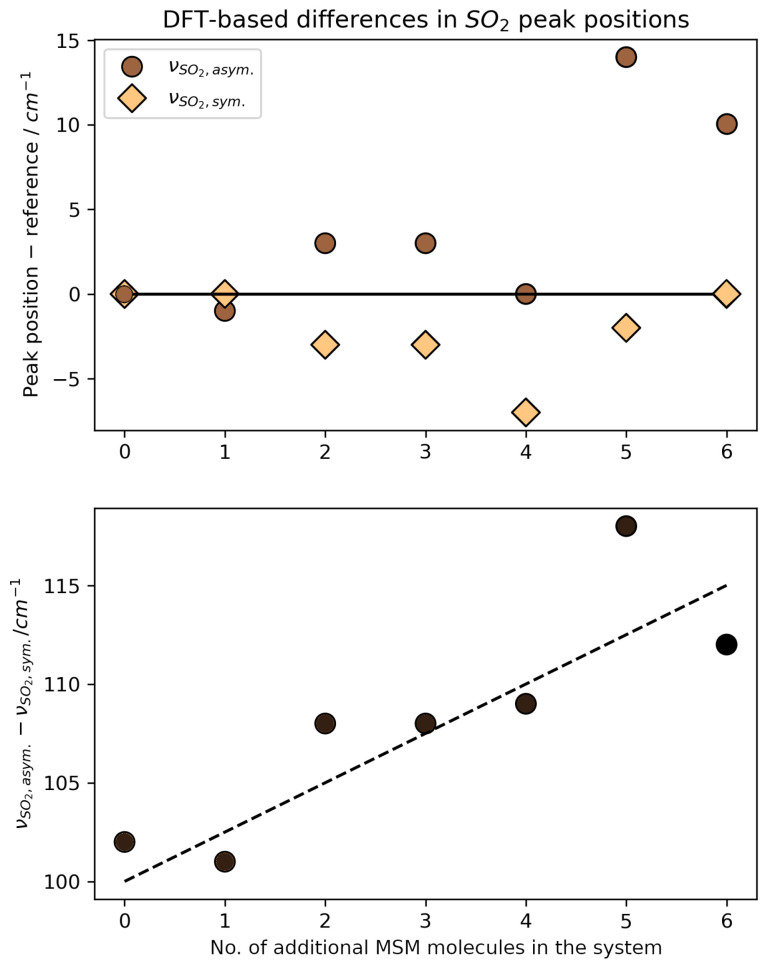
Results of the DFT frequency calculations. The reference system is composed of one central MSM molecule and 33 water molecules (0 additional MSM molecules), and consecutive numbers correspond to additional MSM molecules added to the reference system. Such a procedure simulates an increase in the MSM concentration in a solution. The ONIOM method was used for all calculations. Frequencies were calculated only for the central MSM molecule. **Upper panel:** Differences between the calculated peak positions of SO_2_ stretching modes in concentrated systems and the reference. **Lower panel:** Differences between ν peak positions in consecutive systems indicate the spreading apart of two stretching SO_2_ bands. Simulated DFT-based IR spectra of all systems are available in Supplementary Materials (Figure S10).

To provide a theoretical foundation for these observations, DFT calculations were carried out to simulate the response of MSM SO_2_ vibration modes to an increasing MSM concentration. Using the ONIOM method, a system consisting of a single MSM molecule with its first hydration layer fully closed (33 water molecules) was prepared and optimised. In this model, successive MSM molecules were added to simulate a rise in concentration. The results of these vibration calculations are shown in Figure 5, and the optimised structures of all complexes and corresponding simulated spectra are shown in the Supplementary Materials (Figures S9 and S10).

The computations predict that as MSM concentration increases, the symmetric SO_2_ stretching band should exhibit a red-shift, while the asymmetric SO_2_ stretching band should undergo a blue-shift (Figure 5, upper panel). This opposing shift causes the bands to spread apart (Figure 5, lower panel). In the experimental spectrum, this phenomenon would manifest as the broadening of the bands due to an increase in the intensity of their high and low-energy shoulders. This theoretical prediction is fully consistent with our observations from the normalised transmission spectra (Figure 2) and the ATR-corrected spectra (Figure 4), but contradicts the artefactual red-shifts seen in uncorrected ATR data.

Furthermore, the DFT calculations offer crucial insights into the chemistry of MSM solutions. They confirm that even at high concentrations (e.g., 7:33 MSM:H_2_O, approx. 11 mol kg^−1^), MSM molecules show a strong preference for interacting with water rather than engaging in direct self-interactions. This dominance of MSM:H_2_O interactions is consistent with the high aqueous solubility of MSM. However, it is crucial to consider the strength of these interactions in a relative context. Based on our previous results [12], the interactions between MSM and water are weak when compared to the strong hydrogen bonds that water molecules form among themselves. This is why MSM is considered a weakly hydrated solute; despite its high solubility, it does not form a strongly ordered hydration shell, and water molecules retain a preference for their own cohesive network.

## 3. Materials and Methods

### 3.1. Preparation of Solutions

The solutions were prepared by weight using a Mettler-Toledo XS205 DualRange analytical balance (Columbus, OH, USA) with an accuracy of 0.00001 g. The molalities of MSM (>99%, VWR International, Gdansk, Poland) ranged from 0.0 to 3.5 mol kg^−1^, with the maximum concentration being limited by the solubility of MSM. Solutions were prepared with a step size of 0.05 in the range of 0.0 to 1.0 mol kg^−1^, and with a step size of 0.2 in the range of 1.0 to 3.0 mol kg^−1^. Solutions with molalities above 3.0 mol kg^−1^ were prepared again with a step size of 0.05. The solutions were used for multiple purposes, including measuring density at 25.0 °C using the Anton-Paar DSC5000 densimetre (Graz, Austria), measuring pH at 25.0 °C using a Schott Handylab pH-metre (Mainz, Germany), determining $n_{D}^{25}$ using a Mettler-Toledo digital refractometre (Columbus, OH, USA), and collecting various types of ATR spectra. Due to unavoidable experimental errors, not all solutions were effectively measured using each ATR accessory.

### 3.2. Measurement Parameters

The spectra of all solutions were acquired with an Invenio-R FTIR spectrometre (Bruker, Rosenheim, Germany) that was fitted with three distinct ATR accessories: a single-reflection diamond accessory (Bruker, penetration depth 1.66 μm, $n_{D}^{25}$ = 2.40), as well as six-reflection ZnSe (penetration depth 1.66 μm, $n_{D}^{25}$ = 2.43) and Ge (penetration depth 0.65 μm, $n_{D}^{25}$ = 4.01) liquid cell accessories (Specac). An additional experiment was carried out in the transmission mode (see below). Each spectrum was obtained by averaging 128 individual scans with the resolution of 1 cm^−1^.

In transmission mode, the liquid samples were closed between two CaF_2_ windows without any spacers. This allowed us to measure spectra of aqueous solutions without saturating the detector. However, it was impossible to determine the molar spectra of the sample because its precise thickness varied each time without the control of any spacer. For each measurement, the same set of CaF_2_ windows was used with exactly the same orientation. This minimised the impact of optical artefacts related to discrepancies in the optical path between experiments. These spectra were used to contrast the shapes with the results of ATR correction.

A nitrogen generator (Claind Brezza NiGen LCMS 40-1, Lenno, Italy) produced dry nitrogen, which was used to purge the spectrometre to reduce the impact of carbon dioxide and water vapour. However, a previously reported water vapour subtraction algorithm was used to subtract a small amount of water vapour spectra from each spectrum [16,17]. The collection of at least two atmosphere spectra before and after the actual samples is required to attain optimal performance. An atmosphere sample, as used here, is a measurement made on the IRE element in the absence of any sample.

A water bath temperature controller (Julabo, Seelbach, Germany) was used to keep the temperature of every accessory at a constant 25.0 °C.

As per Equation (1), the water concentration was used to calculate the water-subtraction coefficient for each sample:

(1) $$W_{\mathrm{sub}.\;\mathrm{coef}.}=\frac{C_{\mathrm{water}\;\mathrm{in}\;a\;\mathrm{sample}}}{C_{\mathrm{pure}\;\mathrm{water}}}$$

where *C* stands for molar concentration (mol·dm^−3^). The main vibration bands of MSM and water do not overlap, making the analysis of spectra much easier. Nonetheless, the water spectrum subtraction resulted in an almost straight baseline in the FTIR region characteristic to MSM (1400–900 cm^−1^), as the water spectrum was nearly flat and free of OH-related bands in the examined region.

### 3.3. Software for Spectra Handling and Processing

Spectra were obtained with Bruker Opus 8.7.41. Python 3.11 with the TensorLy library [18] for PFA and PARAFAC [19] spectral decomposition and chemometric analysis were used for all subsequent spectral processing.

The PFA eigenvalue analysis application yielded the number of significant factors, which is referred to as the rank. Chemical reasoning and visual inspection of residual spectra were also employed in the analysis. Although PFA (Malinowski’s Factor Analysis and spectra decomposition algorithm, as described in Ref. [20]) and Multivariate Curve Resolution (MCR) were also initially used for this purpose, the PARAFAC algorithm was ultimately used to perform the decomposition of spectra series. Nevertheless, each time MCR tended to incorrectly identify the first and last spectra in a series as pure factors in our data. The PFA spectra decomposition was limited to the non-negative variant, yet its outcomes were comparable to those of the PARAFAC decomposition. It makes sense because the PARAFAC method was developed on the standard PFA. Because they provided different insights into the systems under analysis, the non-negative and standard unconstrained versions of the spectra decomposition PARAFAC algorithm were both applied. The chemometric analysis was only performed on spectra for MSM concentrations above 0.5 mol·dm^−3^ due to high instrumental noise amplified in molar spectra corresponding to low MSM concentrations.

### 3.4. DFT Calculations

The vibrational frequencies of the free and fully hydrated MSM molecule were calculated using DFT in order to aid in the interpretation of variations in the experimental FTIR spectra. In multiple steps, water molecules were added one after the other to the central MSM molecule to create the full MSM hydration shell of 33 water molecules: (1) Water molecules were added one at a time, optimising the system’s structure and looking for the ideal location for each placement (the minimum energy criterion was used to choose the most optimal structure) for complexes containing up to 10 water molecules; (2) Three water molecules were added to larger complexes in a single steps; (3) Single water molecules were added to the nearly single-layer hydration shell in successive optimisation steps as the shell was about to close. The likelihood of obtaining the optimum geometric structure of the hydration layer increased by this method of calculation preparation. It would take a lot of computing power and produce a random structure of this layer if the entire hydration layer were surrounded by the MSM in one step. (4) To simulate the rise in MSM concentration, up to six extra MSM molecules were added in single steps to the fully hydrated system.

The ONIOM method was applied in steps (2), (3) and (4) where the low layer (the hydration shell and additional MSM molecules) was optimised with a smaller basis set (M06-2X/cc-pVDZ) and the high layer (the central MSM molecule) was optimised with a larger one (M06-2X/aug-cc-pVTZ) [21,22]. In step (1), the same level of theory was applied for MSM (central molecule) and water molecules (M06-2X/aug-cc-pVDZ). This calculation technique made it possible to greatly speed up the process without sacrificing the calculation accuracy for the core molecule. In the final complexes, only the central molecule’s IR spectra were simulated (Freq = ModelModes). The chosen M06-2X DFT functional (the dispersion correction is defined within the method) was developed for organic molecules and weak interaction studies [23].

All calculations were performed in Gaussian 2016 rev.c02 [24]. The vibrations were read from log files and visualised in GaussView5 after corrections suggested on Dr. Joaquin Barroso’s blog [25]. Additional raw computational data, including optimized xyz structures, original Gaussian 2016 log files, and corrected log files (using Dr. Joaquin Barroso’s script), are available in a Zenodo repository at https://zenodo.org/records/17533626, (accessed on 30 October 2025). Computations were carried out using the computers of the Centre of Informatics Tricity Academic Supercomputer & Network.

## 4. Conclusions

This study systematically addressed the challenges of accurately measuring and interpreting the FTIR spectra of the biologically significant molecule, MSM, in aqueous solutions—a critical step for its potential application in quantitative analysis. We have demonstrated that without proper data treatment, particularly ATR correction, spectral data can be misleading, showing artefactual band shifts that obscure the true nature of molecular interactions.

By combining multiple ATR-IREs, transmission measurements, chemometrics, and DFT calculations, we have established a robust approach for obtaining reliable spectral information. Our key findings are as follows:

- For concentration-dependent spectral series, uncorrected ATR-FTIR data are dominated by optical artefacts. In the case of aqueous MSM, these artefacts manifested as a misleading, systematic red-shift in all major bands, obscuring the true chemical information. This finding demonstrates that whilst these minor artefacts may be negligible for routine qualitative analysis, in studies focusing on subtle concentration-dependent effects to interpret molecular interactions, failing to perform a validated ATR correction leads to erroneous spectral interpretation.
- The ATR correction is mandatory to reveal the true molecular interactions. Only after correction did our spectra become consistent with both transmission data and DFT predictions. The corrected data show that increasing MSM concentration primarily causes a broadening of the sulphone vibrational bands, not a shift in their position. This effect is attributed to an increased heterogeneity of the local hydration environments around MSM molecules, a conclusion strongly supported by transmission spectra, theoretical calculations, and previous studies [12] that revealed a complex, multi-population hydration shell structure.
- The choice of ATR internal reflection element (IRE) is critical for successful correction. Our results show that while Ge and diamond crystals yielded spectra that could be reliably corrected to reflect the true chemical phenomena, the correction for ZnSe was insufficient. This provides a practical guideline for future quantitative ATR-FTIR studies on aqueous systems.

Importantly, this work provides a validated methodological blueprint that is an indispensable prerequisite for the development of precise quantitative, ATR-FTIR-based analytical methods that rely on tracking subtle spectral changes. By showing how to eliminate artefacts and isolate the true spectral signature of MSM’s interaction with its environment, we pave the way for creating reliable methods capable of monitoring MSM interactions in complex biological or pharmaceutical matrices.

## Data Availability

The original data presented in the study are openly available in Zenodo repository at https://zenodo.org/records/17533626 (accessed on 30 October 2025).

## Supplementary Materials

The following supporting information can be downloaded at https://www.mdpi.com/article/10.3390/ijms262210953/s1.
