# Supplementary material for: Spectroscopic Probing of Solute–Solvent Interactions in Aqueous Methylsulphonylmethane (MSM) Solutions: An Integrated ATR-FTIR, Chemometric, and DFT Study
Source: Int J Mol Sci. 2025 Nov 12;26(22):10953. doi: 10.3390/ijms262210953 (PMC12652094; doi:10.3390/ijms262210953)
Supplement: Supplementary file 1 [file ijms-26-10953-s001.zip › ijms-3989928-supplementary.pdf]

Supplementary Materials for: Spectroscopic Probing of Solute-Solvent Interactions in Aqueous Methylsulphonylmethane (MSM) Solutions: An Integrated ATR-FTIR, Chemometric, and DFT Study

Aneta Panuszko, Przemysław Pastwa, Paulina Giemza, and Piotr Bruździak*

Department of Physical Chemistry, Gdańsk University of Technology, Narutowicza 11-12, 80-233 Gdańsk, Poland

E-mail: piotr.bruzdziak@pg.edu.pl

S1. ATR spectral series

S1.1. Spectra series before ATR correction corresponding to Ge and ZnSe IREs

Figures S1 and S2 show several series of MSM spectra before ATR correction. These figures correspond to the **left** panel of Figure 1 of the main text.

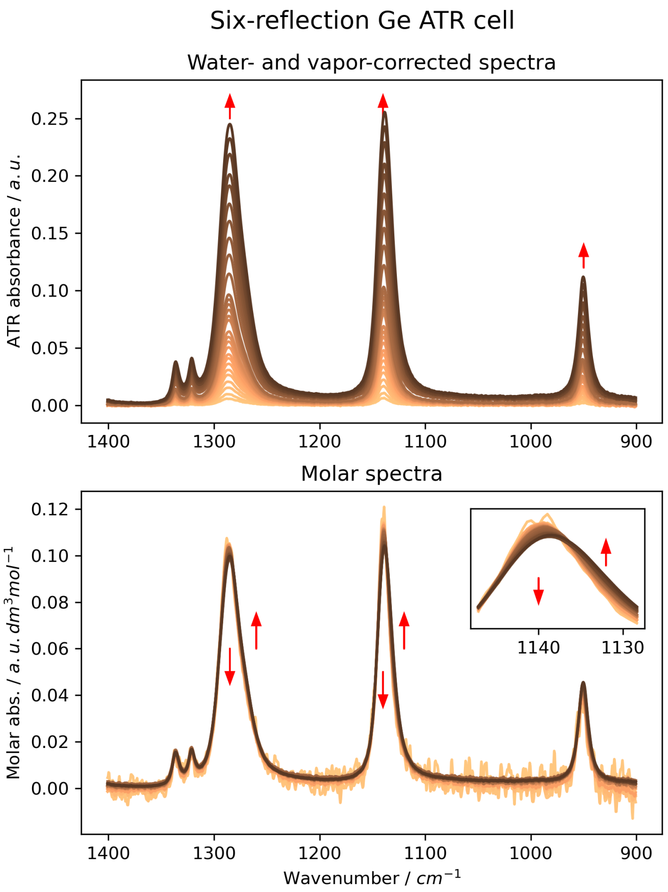

Figure S1: Uncorrected ATR-FTIR spectra of MSM were acquired using a six-reflection **Ge ATR cell**. **Upper panel**: spectra of MSM, devoid of water and water vapour contributions, in the range of SO_2 stretching bands. Arrows indicate an increase in the concentration of MSM in aqueous solutions. **Lower panel**: concentration-corrected molar spectra of MSM. Arrows indicate the main changes in band shapes. **Inset**: an enlarged fragment of the SO_2 band at 1140 cm^{-1}.

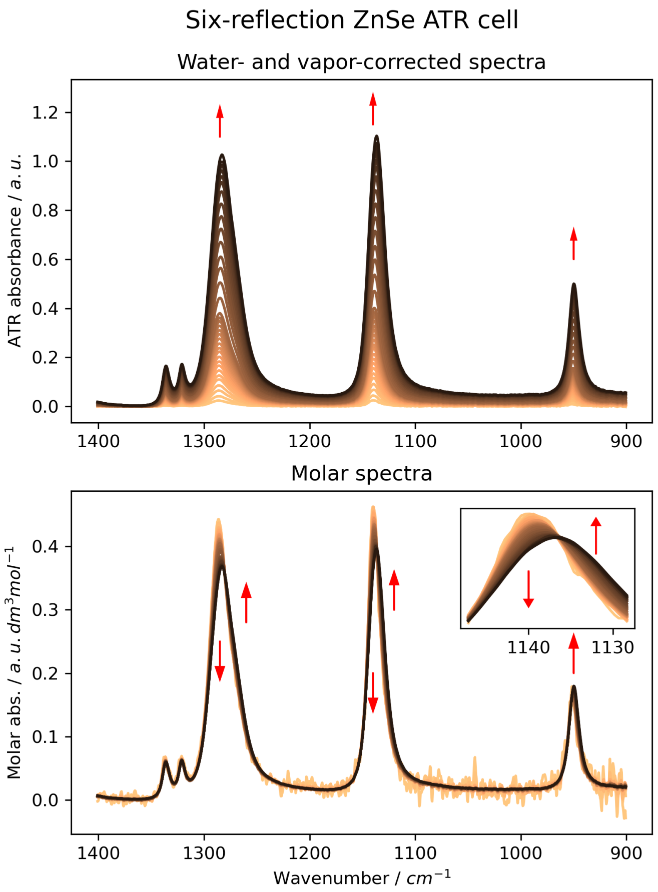

Figure S2: Uncorrected ATR-FTIR spectra of MSM were acquired using a six-reflection **ZnSe ATR cell**. **Upper panel**: spectra of MSM, devoid of water and water vapour contributions, in the range of SO_2 stretching bands. Arrows indicate an increase in the concentration of MSM in aqueous solutions. **Lower panel**: concentration-corrected molar spectra of MSM. Arrows indicate the main changes in band shapes. **Inset**: an enlarged fragment of the SO_2 band at 1140 cm^{-1}.

S1.2. Spectra series after ATR correction corresponding to Ge and ZnSe IREs

Figures S3 and S4 show several series of ATR-corrected MSM spectra. These figures correspond to the **right** panel of Figure 1 of the main text.

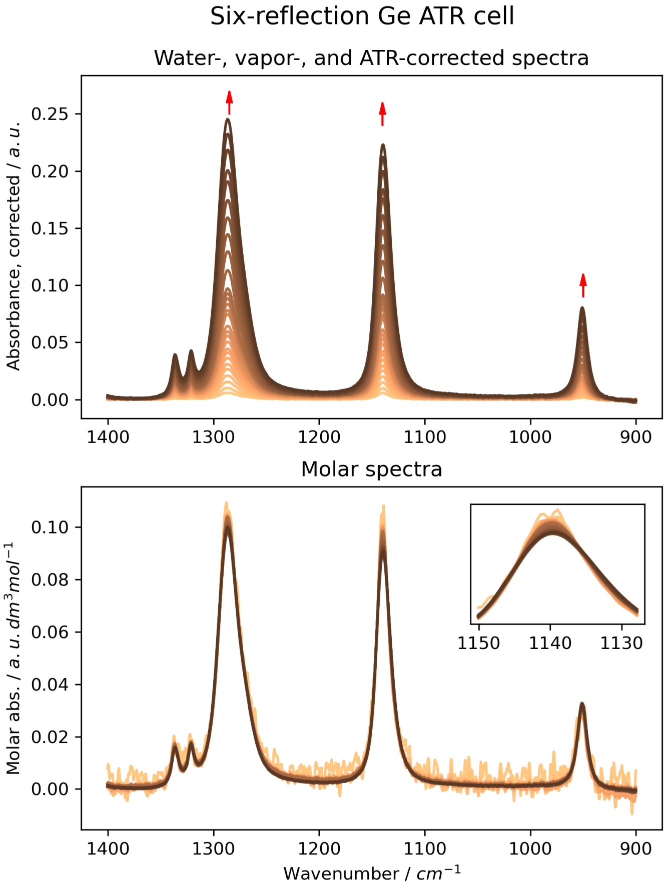

Figure S3: ATR-corrected spectra of MSM in aqueous solutions (0.0–3.0 $mol \cdot dm^{-3}$) were acquired using a six-reflection Ge ATR cell. **Upper panel**: water- and vapour-corrected spectra of MSM in the range of SO_2 stretching bands. Arrows indicate an increase in the concentration of MSM in aqueous solutions. **Lower panel**: concentration-corrected molar spectra of MSM. Arrows indicate the main changes in band shapes. **Inset**: an enlarged fragment of the SO_2 band at 1140 cm^{-1}.

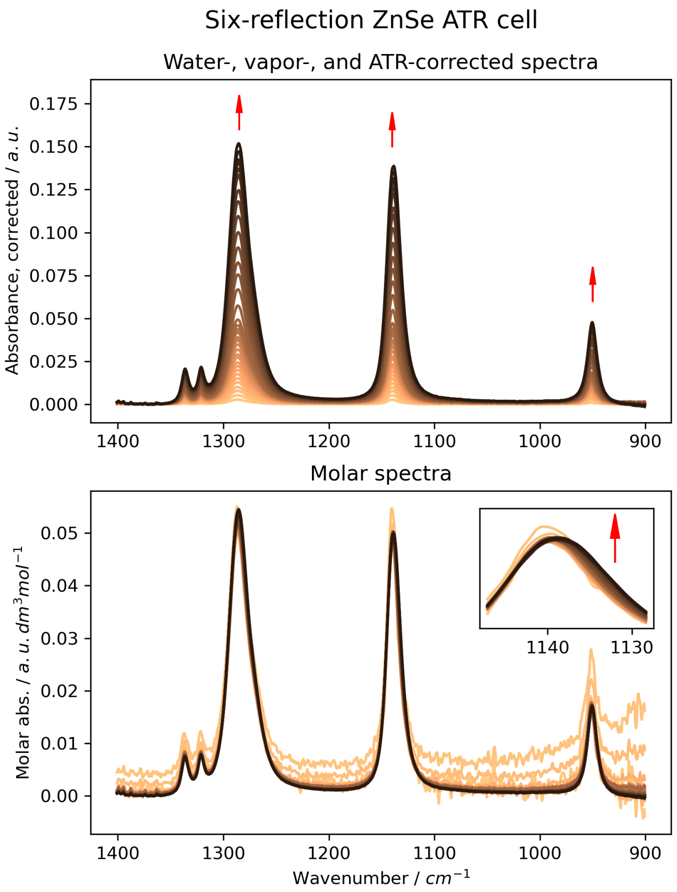

Figure S4: ATR-corrected spectra of MSM in aqueous solutions (0.0–3.0 $mol \cdot dm^{-3}$) were acquired using a six-reflection ZnSe ATR cell. **Upper panel**: water- and vapour-corrected spectra of MSM in the range of SO_2 stretching bands. Arrows indicate an increase in the concentration of MSM in aqueous solutions. **Lower panel**: concentration-corrected molar spectra of MSM. Arrows indicate the main changes in band shapes. **Inset**: an enlarged fragment of the SO_2 band at 1140 cm^{-1}.

S2. Detailed Chemometric Analysis Strategy and Results

S.2.1. Negative or non-negative spectra decomposition?

The number of factors used in the decomposition of spectra series is a matter of debate. The eigenvalue analysis is often inconclusive and should only be used as a reference or as a starting point for additional analysis, possibly based on residual inspection. The question "How many factors?" may not always be a good one because what is expected is a strict integer. It would be more appropriate to ask "How does the system react?" given the kind of data this paper presents, as this could be adequately addressed by two, three, or more factors. As a result, the number of factors that make up the system need not be restricted to the spectra of "pure" components; instead, it can be more abstract and include baseline drift, non-instrumental noise (such as vapour contribution), and any change caused by an external trigger (in our case, concentration).

To reduce the quantity of those abstract factors in our research, we carried out a number of pre-treatment procedures. **1)** Despite being extremely minimal in the examined area, the noise caused by water vapour was removed with a previously described algorithm [16]. **2)** In a similar fashion, the water content was also removed in the MSM absorption region, resulting in a flat baseline, even though the water bands did not overlap significantly in the main region. As a result, two spectra-affecting factors were eliminated without the need for artificial smoothing or baseline corrections. **3)** Furthermore, a series of molar spectra devoid of a strong and abstract concentration factor was generated by dividing all spectra by their molar concentrations. The elimination of these three factors allowed that the subsequent analysis was concentrated on those significant chemical or physical variables that were closely linked to the solution's chemistry or, as suspected, optical effects.

For factor extraction, the standard chemometric spectra decomposition (free of non-negativity constraints) and non-negative pathways of chemometric spectra decomposition

can be chosen. Non-negative variants of PFA, PARAFAC, MCR, and other methods will favor factors that are as similar to a spectrum as possible and show no negative bands. Because bands of these spectrum-like factors can be assigned to specific chemical groups or transitions, this method of analysis appears to be simpler and easier to understand. Conversely, the standard method of decomposition (without the non-negativity constraint) can yield factors that resemble derivative-like spectra or difference spectra. Although these abstract factors are difficult to understand at first, they provide the same information as non-negative factors. According to our experience, non-negative spectra decomposition methods yield a higher number of factors than the standard ones, but provide the same information. These higher numbers do not indicate the true meaningful number of factors, but rather relate to the method's attempt to solve a specific mathematical problem under given constraints. Here, it is important to keep in mind that factors—even those that are non-negative and resemble real spectra—are merely non-unique outcomes of mathematical operations, and their significance needs to be understood in light of the particular problem at hand.

S.2.2. Results of chemometric analysis – spectra without ATR correction

Results of PARAFAC decomposition (standard and non-negative) of ATR spectra series before ATR correction are presented in Figures S5 and S6 for Ge and ZnSe IREs, respectively (left panels for standard and right panels for non-negative variants of PARAFAC, respectively). These figures correspond to Figure 3 of the main text.

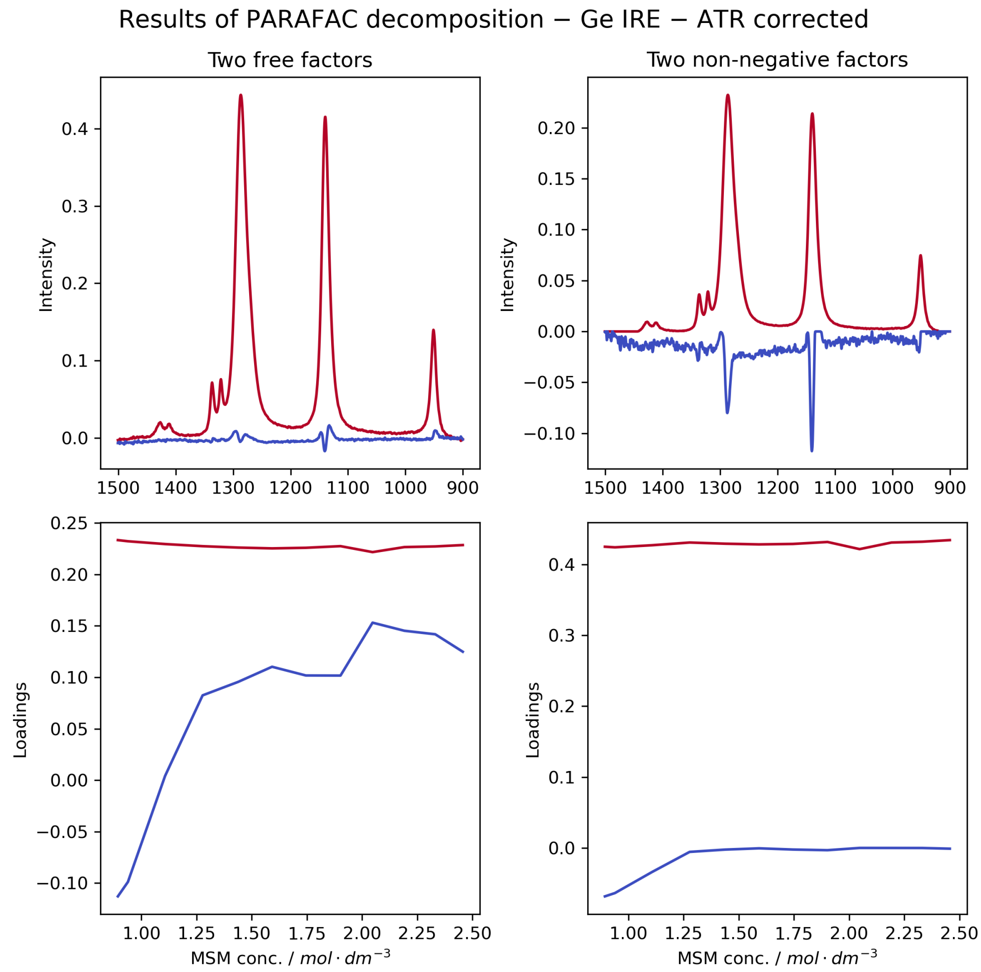

Figure S5: Results of PARAFAC decomposition of ATR-FTIR spectra acquired with a six-reflection G e A TR c ell. W e o btained t he f actors a nd t heir l oadings u sing t he f ree, or unconstrained (left panels), and non-negative, or constrained, variants of the decomposition algorithm.

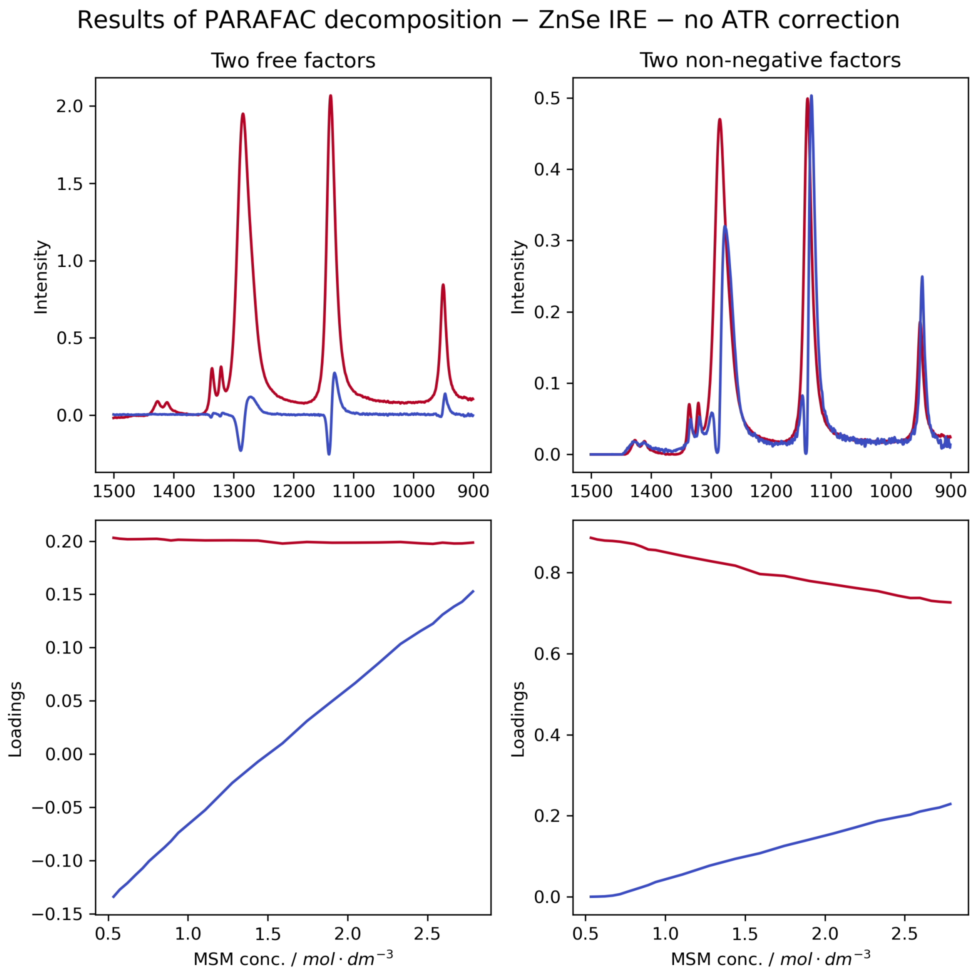

Figure S6: Results of PARAFAC decomposition of ATR-FTIR spectra acquired with a six-reflection ZnSe ATR cell. We obtained the factors and their loadings using the free, or unconstrained (left panels), and non-negative, or constrained, variants of the decomposition algorithm.

Using the PARAFAC algorithm, the molar ATR spectra series can be split into two significant factors. While it may be tempting to try to obtain three different "pure" component spectra, especially using the non-negative alternative, we believe it is preferable to limit the number to two and allow factors to take negative values.

A clear average MSM spectrum could be identified as one of the two factors (red line), with the second factor (blue) corresponding to changes in the MSM spectrum's shape. This second factor bears a distinct resemblance to a differential spectrum. The S-like shape of this factor is suspiciously similar to the refraction index-wavelength relationship inherent to

an ATR spectrum, i.e., it could be attributed solely to the optical effects native to the ATR method rather than to the real chemistry of the solution.

The constrained, non-negative PARAFAC decomposition creates an artificial picture of the same situation. While the average MSM spectrum appears to be almost the same as in the case of standard decomposition, the second one (blue) clearly displays anomalies resulting from mathematical constraints. It is, in fact, a very similar factor to the blue one on the left panel, with all negative bands' signs reversed. In this scenario, it could be tempting to decompose the series into three non-negative components, potentially masking the mentioned effect. However, the third factor in non-negative decomposition would be just a mathematical solution that attempts to make up for the negative portion of one of the factors in standard decomposition.

At this point, we can conclude that the changes observed in ATR-FTIR spectra were primarily the result of an optical artifact rather than shifts resulting from real MSM–H_2O or MSM–MSM interactions in solution, even though the measurements were made for aqueous solutions with a slight variation in n_D^{25}.

S.2.3. Results of chemometric analysis – spectra after ATR correction

Results of PARAFAC decomposition (standard and non-negative) of ATR spectra series after advanced ATR correction are presented in Figures S7 and S8 for Ge and ZnSe IREs, respectively (left panels for standard and right panels for non-negative variants of PARAFAC, respectively). These figures correspond to Figure 4 of the main text.

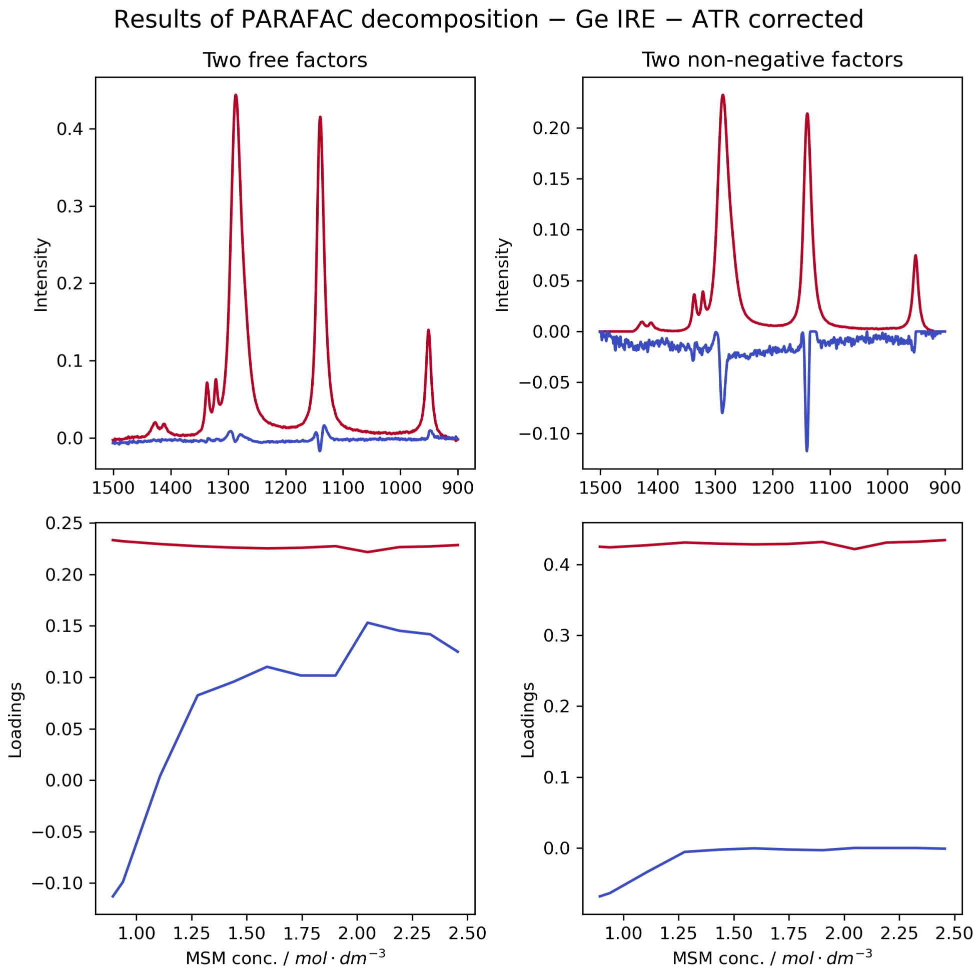

Figure S7: Results of PARAFAC decomposition of ATR-FTIR spectra corrected with an advanced ATR correction algorithm. As in Figure S5, factors and their loadings were found using both free (left panels) and non-negative (right panels) versions of the decomposition algorithm. In the right panels, we have reversed the signs of the second factor and its loading (blue) to better compare with the results of free decomposition. Such a change has only a visual meaning and gives no net change in the mathematical meaning of the blue factor.

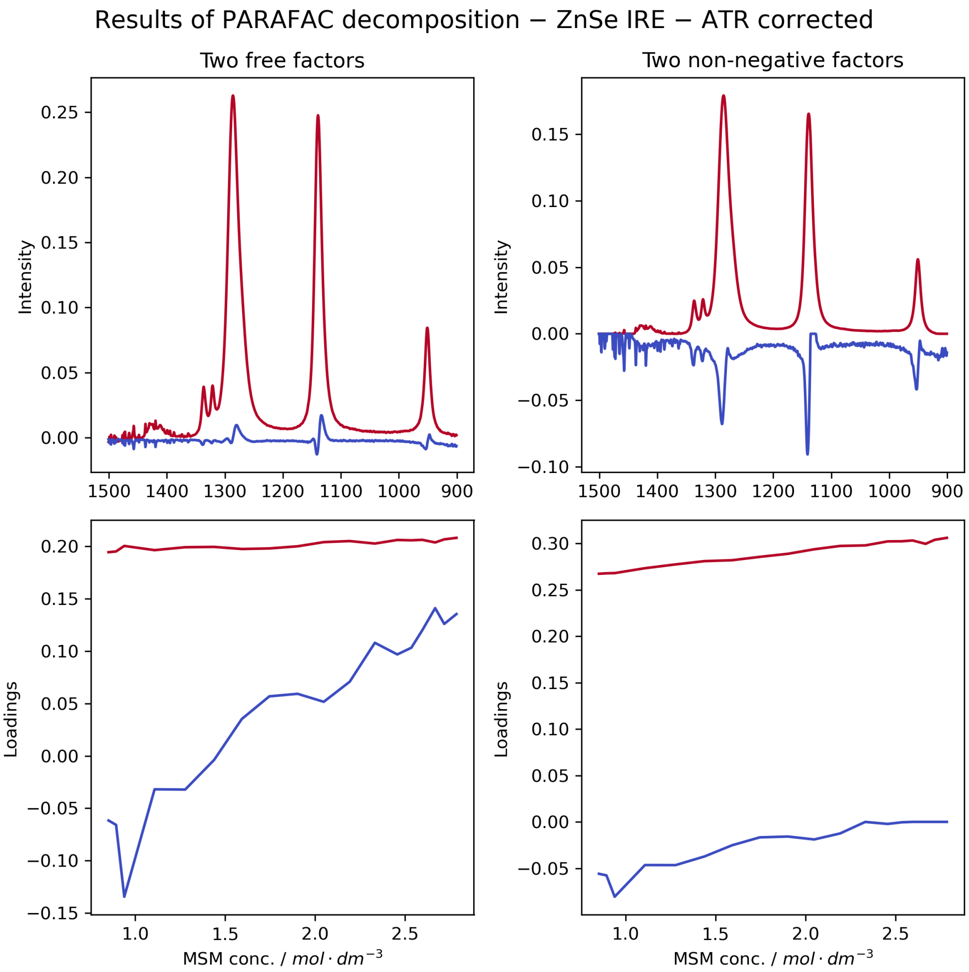

Figure S8: Results of PARAFAC decomposition of ATR-FTIR spectra corrected with an advanced ATR correction algorithm. As in Figure S6, factors and their loadings were found using both free (left panels) and non-negative (right panels) versions of the decomposition algorithm. In the right panels, we have reversed the signs of the second factor and its loading (blue) to better compare with the results of free decomposition. Such a change has only a visual meaning and gives no net change in the mathematical meaning of the blue factor.

The shape and direction of changes in the spectra series were completely altered by the ATR correction (see Figure 4 of the main text, and Figures S7 and S8). Like with transmission spectra, the most important aspect is that the shape of the second, difference-like factor indicates that MSM peaks remain in the same positions. According to standard unconstrained PARAFAC, in the cases of Ge and Diamond IREs, the asymmetric SO_2 stretching band (1285 cm^{-1}) intensifies slightly its signal in the band's high energy r egion. The behav-

ior of the symmetric stretching band (1138 cm^{-1}) can be interpreted as a change in width (a W- or M-like difference peak shape). These changes are quite similar to those recognizable in the transmission series, at least qualitatively. However, the ZnSe IRE case shows weaker but similar changes compared to the case without the ATR correction, leading us to conclude that the correction was probably insufficient. Therefore, ZnSe would not be the preferred crystal in spectra series that are concentration-dependent.

Although the ATR correction resulted in agreement between ATR and transmission spectra (at least in the case of diamond and Ge), a clear unfavorable effect can be observed. An additional baseline drift was introduced by the correction, particularly for samples with low MSM concentrations. It was replicated in PARAFAC factors, particularly in the non-negative algorithm case, where obtaining a typical factor-like spectrum is impossible due to the baseline shift. Instead, the second non-negative factor has derivative-like shapes that are similar to those obtained with the standard variant of the algorithm. Technically, the factors are not negative, but the introduced baseline drift mathematically breaks the non-negativity.

All of this may suggest that when dealing with concentration-dependent series like the ones this paper presents, the ATR correction—while not flawless or without issues—must be carried out.

S3. DFT results

Structures of $MSM - 33 \cdot H_2O - n \cdot MSM$ complexes presented in Figure S9 were optimised according to the procedure presented in the main paper. n denotes the number of additional MSM molecules in the system.

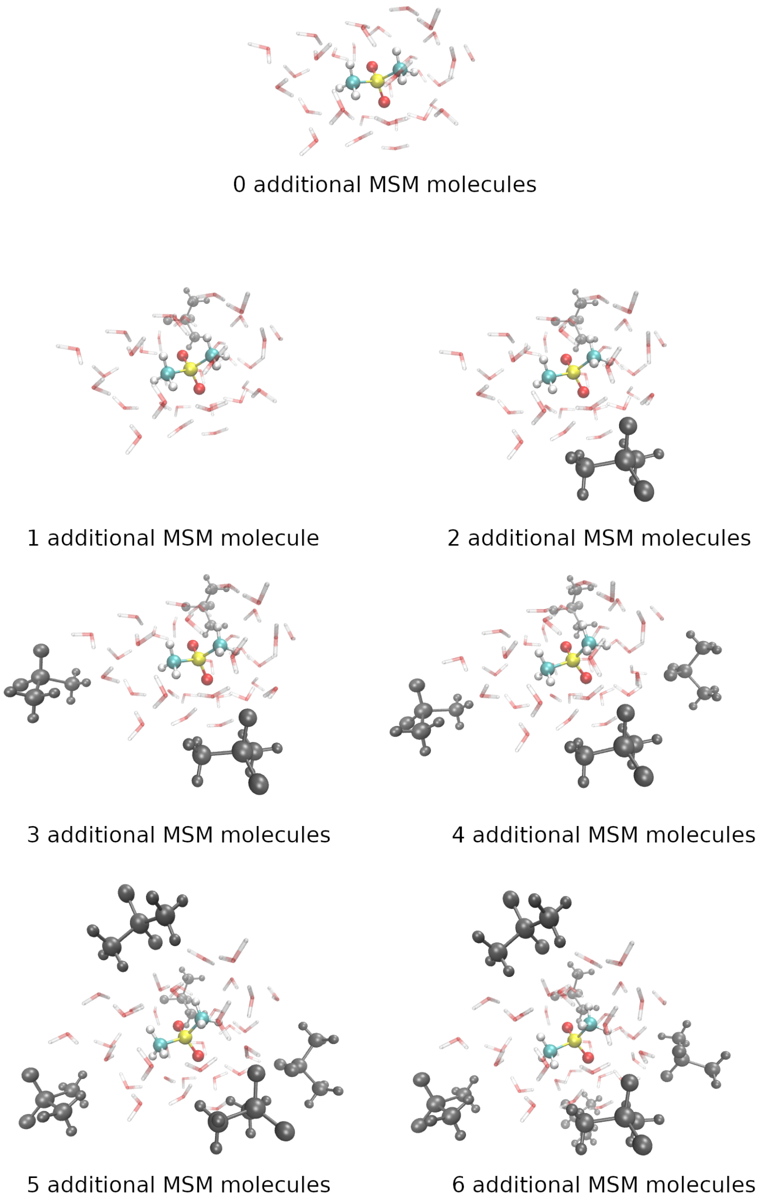

Figure S9: Optimised structures of $MSM - 33 \cdot H_2O - n \cdot MSM$ complexes. The central MSM molecule is colored as follows: red – oxygen, yellow – sulphur, cyan – carbon, light gray – hydrogen. Water molecules are represented by transparent stick structures. The additional MSM molecules are gray.

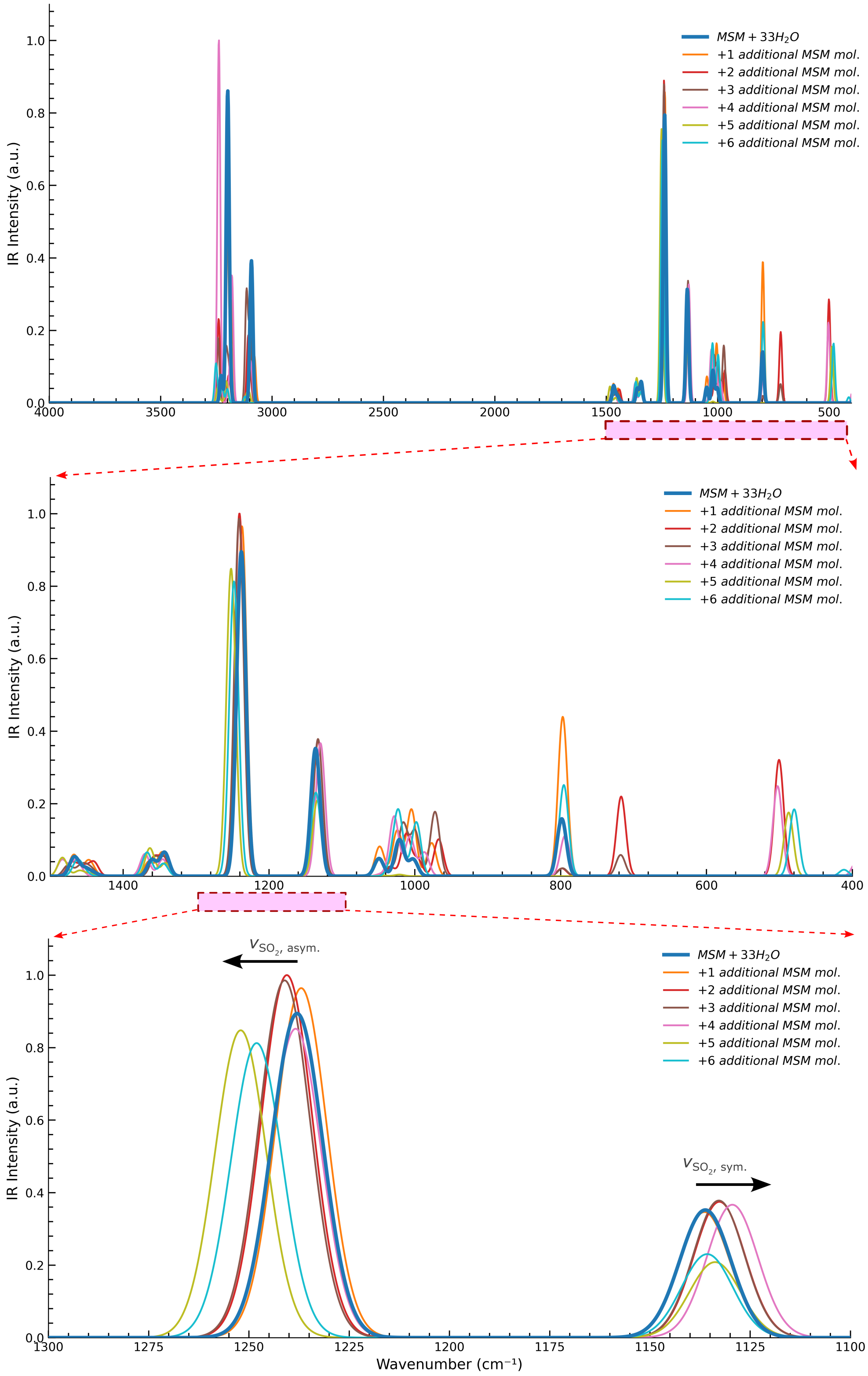

Figure S10: Simulated DFT-based IR spectra of $MSM - 33 \cdot H_2O - n \cdot MSM$ complexes (Gaussian peaks with a FWHM of 15 cm^{-1}).
